# A Novel Atomically Resolved Scanning Tunneling Microscope Capable of Working in Cryogen-Free Superconducting Magnet

**DOI:** 10.3390/mi14030637

**Published:** 2023-03-11

**Authors:** Tao Geng, Jihao Wang, Wenjie Meng, Jing Zhang, Qiyuan Feng, Yubin Hou, Qingyou Lu

**Affiliations:** 1 High Magnetic Field Laboratory, Hefei Institutes of Physical Science, Chinese Academy of Sciences, Hefei 230031, China; 2 University of Science and Technology of China, Hefei 230026, China; 3 Anhui Province Key Laboratory of Condensed Matter Physics at Extreme Conditions, High Magnetic Field Laboratory, Chinese Academy of Sciences, Hefei 230031, China; 4 High Magnetic Field Laboratory of Anhui Province, Hefei 230031, China; 5 Anhui Laboratory of Advanced Photon Science and Technology, University of Science and Technology of China, Hefei 230026, China; 6 Hefei National Research Center for Physical Sciences at the Microscale, University of Science and Technology of China, Hefei 230026, China

**Keywords:** scanning tunneling microscope, cryogen-free superconducting magnet, atomic-resolution imaging, low temperature, compact tip–sample loop

## Abstract

We present a novel homebuilt scanning tunneling microscope (STM) with atomic resolution integrated into a cryogen-free superconducting magnet system with a variable temperature insert. The STM head is designed as a nested structure of double piezoelectric tubes (PTs), which are connected coaxially through a sapphire frame whose top has a sample stage. A single shaft made of tantalum, with the STM tip on top, is held firmly by a spring strip inside the internal PT. The external PT drives the shaft to the tip–sample junction based on the SpiderDrive principle, and the internal PT completes the subsequent scanning and imaging work. The STM head is simple, compact, and easy to assemble. The excellent performance of the device was demonstrated by obtaining atomic-resolution images of graphite and low drift rates of 30.2 pm/min and 41.4 pm/min in the X–Y plane and Z direction, respectively, at 300K. In addition, we cooled the sample to 1.6 K and took atomic-resolution images of graphite and NbSe_2_. Finally, we performed a magnetic field sweep test from 0 T to 9 T at 70 K, obtaining distinct graphite images with atomic resolution under varying magnetic fields. These experiments show our newly developed STM’s high stability, vibration resistance, and immunity to high magnetic fields.

## 1. Introduction

Since the invention of the scanning tunneling microscope (STM), it has been used to observe the atomic structure and the local electronic density of state on the surface of materials [1] and obtain spectral information to analyze the energy band structure [2]. Hence, it has great significance and application in the fields of materials science, biological science [3,4], condensed matter physics, and other areas. At present, more and more STMs are being advanced to work in high magnetic fields [5,6,7] and low-temperature environments [8,9], which has significant scientific significance for studying the quantum Hall effect [10,11], vortex issues [12], Ising superconductor [13], electronic nematicity [14], and other cutting-edge problems in physics.

However, STMs tend to be very sensitive to external vibration and acoustic noise [15,16]. Hence, most high magnetic field STMs working in low-temperature conditions are placed in wet superconducting magnets, whose superconducting coils are entirely immersed in liquid helium to maintain the superconducting characteristic [17,18]. Although the system provides a quiet and low-vibration environment, it requires a large amount of liquid helium to support the operation. Moreover, the recovery, purification, and reuse of helium are also time-consuming and laborious. In contrast, cryogen-free magnets, which are gradually being developed, use a closed-loop helium expansion cycle to sustain a low-temperature environment for the magnet coil [19,20], thus the device takes little effort to maintain and operate. However, such a setup requires a cryocooler that generates severe vibrations when running, which is very detrimental to the atomically resolved imaging of the STM. The development of STMs to work in a cryogen-free magnet system is a great challenge due to the extremely high requirements for the anti-vibration and rigidity properties of the STM head and the absorption of vibration from the external environment [21].

Our group has previously designed and built several STMs for use in strong magnetic fields [22,23] and low-temperature environments [24], all of which have excellent imaging performance. Their STM head structure is a double slider push structure based on the SpiderDrive [25] to drive the tip or sample into the tunneling-current-junction area to enable high-precision scanning imaging. However, this structure has very high requirements for the processing accuracy and fit of the double sliders, which require high coaxiality to ensure that the large slider of the SpiderDrive will not generate a lateral force on the small slider fixed at the bottom of the scanning tube when we perform a tip-withdrawal operation. Therefore, in constructing our later 30 T hybrid magnet STM [26], we designed a single shaft structure based on the SpiderDrive principle. Since it uses a single piezoelectric tube (PT) to achieve the coarse approach and precise scanning, the lateral size of the PT cannot be too large because of the high stability and low drift rate required for scanning. Therefore, the driving force of the coarse approach is not very large. Moreover, a pulse signal higher than the reference potential applied on the PT to drive the shaft to withdraw at the low temperature will inevitably cause a tip-to-sample crash.

Herein, we present a new, simple, compact STM whose head is mainly made of non-metallic materials. Two PTs and a sapphire frame are nested and positioned coaxially, in which the inner PT responsible for scanning is nested within the sapphire frame, and the sapphire frame is nested inside the outer PT used for the coarse approach. The overall structure is compact and vibration-resistant, with only a single shaft. Furthermore, we integrated this design into a newly designed probe rod placed in a cryogen-free superconducting magnet for a series of tests. First, an atomic-resolution image of graphite and drift data were obtained at room-temperature 300 K. Then, we acquired the atomically resolved images of graphite and NbSe_2_ at 1.6 K by cooling the system. In addition, we performed a measurement of the tunneling current spectrums, and the noise level was sufficiently low. Finally, testing in a sweeping magnetic field from 0 T to 9 T was carried out at 70 K. The clear atomic-resolution images of graphite obtained in these tests showed our STM’s strong immunity to magnetic fields and high vibration resistance. The STM head’s small longitudinal dimension of 45 mm gave it the potential to be used to develop a rotatable STM for use in high magnetic fields.

## 2. STM Head Design

**Figure 1:**
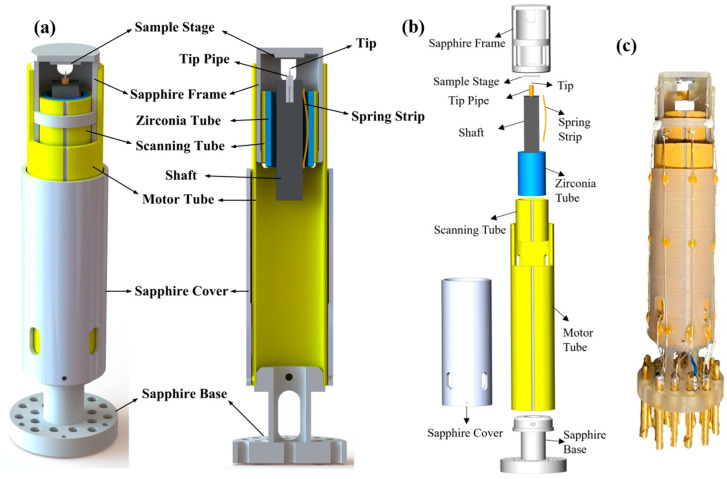
(**a**) Front schematic and sectional view of the STM head unit. (**b**) Exploded view of the STM head unit, in which the main components are indicated. (**c**) Photograph of the STM head unit.

The entire STM head unit consists of seven parts, as shown in its schematic diagram and exploded drawing in Figure 1a,b. (1) A sapphire base serving as the hanging point and fixed end, on which there are 20 holes with a diameter of 1.5 mm used to glue pins to connect the signal wires. (2) A PT with an outer electrode split into four quadrants and one complete inner electrode (EBL#3 type from EBL Products Inc., East Hartford, USA), called the motor tube, with 10.1 mm outer diameter (OD), 0.55 mm wall thickness (WT), and 36 mm length. It has a large notch of 8.5 mm length and 8 mm width starting at the dividing-electrode line and two slotted holes of 4 mm length and 1.5 mm width in the middle of the two electrode quadrants opposite the large notch at one end. The notch and two slotted holes were both drilled by a small grinding head. (3) A sapphire frame, 8.93 mm OD and 14 mm long, on who’s top a sample stage is mounted, with a large window of 6.9 mm width milled on the front side and two slotted holes of 1.4 mm width and 4 mm length milled on the back side. (4) A PT with length 9.5 mm, OD 7.59 mm, and WT 0.55 mm (EBL#3 type from EBL Products Inc.), called the scanning tube, with a four-quadrant outer electrode and one complete inner electrode. (5) A zirconia tube with 6.45 mm OD and 10mm length, with the inner wall highly polished. (6) A spring strip of beryllium copper, with a thickness of 0.2mm. (7) A 15 mm long shaft with a 3.5 mm by 3.5 mm cross-section, made of tantalum, surface mirror-polished, and having a tip pipe installed at the upper end.

The seven components are assembled in a highly concentric manner. The sapphire base is coaxially glued at the bottom of the motor tube with H74 epoxy (from Epoxy Technology Inc. Billerica, USA). The sapphire frame is nested inside the motor tube, and its upper end is fixed to the top of the motor tube’s inner wall with glue. It is necessary to ensure that the 8 mm wide notch of the motor tube corresponds to the 6.9 mm wide window of the sapphire frame and that the two 4 mm long slotted holes of the motor tube coincide with two slotted holes of the sapphire frame, respectively. The scanning tube is nested inside the sapphire frame, glued together at their lower ends flush. Inside the zirconia tube, the shaft is clamped by the spring strip to move smoothly along the axis with the shaft’s upper end with the tip pointed toward the sample stage. This tube and shaft assembly is inserted into the scanning tube and glued at its upper end. The two outer electrodes on the front of the scanning tube are connected by a 0.04 mm Pt wire with H20E silver conductive epoxy (from Epoxy Technology Inc.) from the window of the sapphire frame and the large notch of the motor tube. The other two outer and inner electrodes on the back are linked through the 4 mm long slotted holes of the sapphire frame and motor tube. The large notch of the motor tube and the large window of the sapphire frame make the operation of the tip and the exchanging of samples more convenient. The STM head unit is simple, compact, and easy to install. Figure 1c is a photo of the assembled STM head unit.

The tip pipe is a capillary tube made of copper with an outer diameter of 1.0 mm, an inner diameter of 0.3 mm, and a length of 3.6 mm. The tip pipe was inserted into the top of the shaft, where a hole had been drilled with a diameter of 1.05 mm and a depth of 3 mm, and then glued with H74 epoxy. The tip used in the experiment was a hand-cut 90:10 Pt-Ir wire (annealed, from Goodfellow Cambridge Limited) with a diameter of 0.25 mm; the angle between the Pt-Ir wire and the scissors is between 35° and 45°. Pt-Ir tip is not easy to oxidize in air, relatively stable, and easy to prepare. The tip was glued into the tip pipe by means of silver conductive paint (which can be dissolved in acetone) to ensure its stability at low temperatures. The sample stage is a 4 mm × 4 mm sapphire sheet with a thickness of 0.5 mm. After the sample was mounted, it was also fixed to the top of the sapphire frame by silver conductive paint. The bias voltage was applied to the tip by a 0.04 mm Pt wire, and the sample was also grounded via a Pt wire.

When the STM head performs the coarse approach, a slow probe voltage signal is applied to the inner electrode of the scanning tube to confirm that the distance between the tip and sample is safe, and then a pulse signal is applied to the four outer electrodes of the motor tube whose inner electrode is grounded. The waveform of the signal, referred to in Ref. [25], generates the inertial force to drive the shaft’s forward displacement. Continue this process until the tip on the shaft reaches the tunneling-current-junction area. At this time, the four outer electrodes of the motor tube are directly grounded. The scanning voltage signal is applied on the four external electrodes of the scanning tube, and a slowly changing voltage is used on the inner electrode to control the tiny distance finely between the tip and sample to complete the acquisition of the tunneling current. Since the voltage signals of the motor tube and scanning tube are applied separately, when the tip withdraws at a low temperature, the motor tube can receive a larger pulse voltage from a high potential on the outer electrodes to work more smoothly, which cannot be performed by the STM in Ref. [26].

Since the scanning tube is nested inside the motor tube, the longitudinal dimension can be small while ensuring a certain motor driving force. The mechanical disturbance and electronic interference of the motor tube cannot enter the tip–sample loop, which is completely fixed at the free end of the motor tube. The tip–sample mechanical loop is very compact, and the weight of the shaft inside the scanning tube makes the scanning structure more rigid, stable, and vibration resistant. The entire STM head was made of non-metallic materials except for the shaft and spring strip so that it can be well used in environments with strong magnetic fields. Compared with a metallic head, the non-metallic STM head can effectively prevent the eddy current phenomenon in high magnetic fields. Since the shaft is an inertial slider that requires a certain weight, we used tantalum metal to make it, which is non-magnetic and can also be well-compatible with high magnetic fields. The scanning head features a sapphire frame for better cooling at low temperatures. The small tip–sample loop and highly symmetrical design result in more downward thermal drift. The STM head’s small horizontal and vertical dimensions make it easier to integrate into narrow spaces.

## 3. System Design

**Figure 2:**
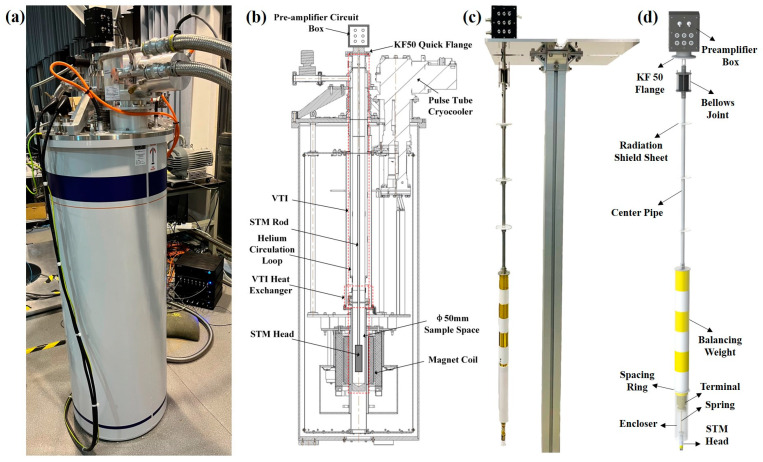
(**a**) Photo of the magnet system with the STM rod inserted. (**b**) Schematic diagram of the section of the magnet system with the STM rod inserted. (**c**) Photograph of the STM rod. (**d**) Schematic of the STM rod, in which the main parts are indicated.

We used a cryogen-free superconducting magnet (model: TeslatronPT) purchased from Oxford Instruments. The middle part of the magnet is an integrated variable temperature insert (VTI), in which the central tube, usually referred to as the sample space, has a diameter of 50 mm, into which an STM rod can be inserted from the top via a KF50 quick flange. In the VTI, a helium circulation loop, which is completely separate from the sample space, controls the rate and magnitude of system cooling by adjusting the flow through a needle valve. In addition, a VTI heat exchanger with a temperature sensor and heater monitors and controls the VTI temperature through a PID feedback loop. The sample space is filled with static helium exchange gas to maintain good thermal exchange with the VTI heat exchanger, thus achieving variable temperatures from 1.6 K to 300 K for the inserted STM head and sample. Outside the bottom of the sample space is a superconducting coil of the magnet cooled by a cryostat. In this experiment, when the STM rod is inserted into the sample space, the STM head is located precisely in the central area of the magnet coil, which generates a magnetic field from 0 T to 9 T. A photo and schematic of the whole system appear in Figure 2a,b.

The picture and three-dimensional rendering of the STM rod are shown in Figure 2c,d. Its structure mainly includes a preamplifier box, a KF50 flange, a bellows joint, a center pipe, three radiation shield sheets, a balancing weight, a spacing ring, a terminal, a vibration isolation spring, an encloser, and the STM head, which is similar to the STM probe previously published [22]. Our homemade STM preamp circuit is built into the preamplifier box, with a feedback resistor of 100 MΩ and a bandwidth of 200 Hz. Between the KF50 flange and the preamplifier box, there is a self-made electrical feedthrough for transmitting electrical signals while sealing the sample space, the structure of which is discussed in Ref. [27]. Considering the light weight of the sapphire STM head, the signal cables connected from the STM head to the underside of the terminal are enameled-silver wires of a 0.07 mm diameter. On the outside of the STM head, an encloser screwed to the top of the terminal ensures the connected enameled-silver wires do not touch the inner wall of the sample space to reduce interference. New cables on top of the terminal are connected to the electrical feedthrough at the bottom of the preamplifier box through the central hole of the balancing weight and center pipe. Above the terminal, a spacing ring made of Teflon is fixed, on which several axial holes are drilled at the edge to increase its elasticity.

The STM rod uses a two-stage passive isolation mechanism to attenuate vibrations. The first stage is a soft welded bellows attached above the center pipe, below which is a balancing weight formed by alternating connections of copper and Teflon to prevent heat transfer, resulting in a lower natural frequency. The second stage has the STM head suspended above the bottom of the terminal via the vibration isolation spring with a 3.5 mm diameter and 90 mm length, wound by 0.15 mm diameter beryllium copper wire, realizing an eigenfrequency of about 2 Hz. The STM rod is mounted to the magnet by the KF50 flange; the clamp used is a plastic clamp, and the sealing ring is a polymer gasket to achieve electrical isolation from the magnet. The ground connections of the STM head and STM rod are connected to a particular ground wire that is a thick copper pillar buried in the ground and which is not the ground of the magnet system, eliminating some electromagnetic interference generated by the magnet.

The STM controller we used was homemade and based on a NI data acquisition card of the model PXI-7851R, which enables the coarse approach, scanning, imaging, and data acquisition. The real-time control software used was written by us in LabVIEW. The controller and power module were placed 1.5 m away from the magnet’s center to prevent interference from the magnet field.

## 4. Performance Test

**Figure 3:**
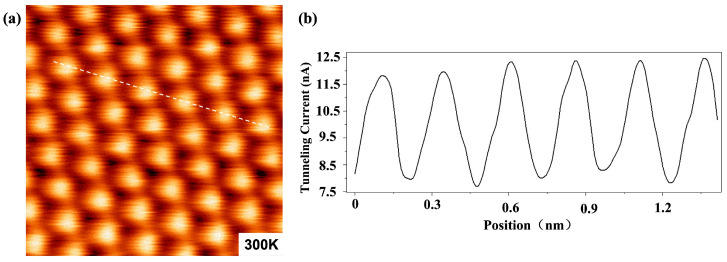
(**a**) Atomic-resolution STM image of HOPG taken in constant height mode at 300 K in a cryogen-free superconducting magnet. Scan area = 1.7 nm × 1.7 nm, bias voltage = + 200 mV, and average tunneling current = 10 nA. (**b**) Cross-sectional profile along the white dashed line.

First, we scanned the surface of highly orientated pyrolytic graphite (HOPG, grade SPI-2 from Structure Probe, Inc., West Chester, USA) with the STM at a VTI temperature of 300 K after the completion of the coarse approach. A clear atomic-resolution image was obtained, as seen in Figure 3a, demonstrating our STM’s precise imaging performance. Figure 3b is the tunneling current spectrum along the white dotted line in Figure 3a, showing high smoothness and signal-to-noise ratio. During the imaging, a bias voltage of +200mV was applied to the tip, and the sample was grounded. The high stability and high accuracy of the STM at room temperature were confirmed by the high-quality imaging attributable to the high compactness and rigidity of the STM head.

**Figure 4:**
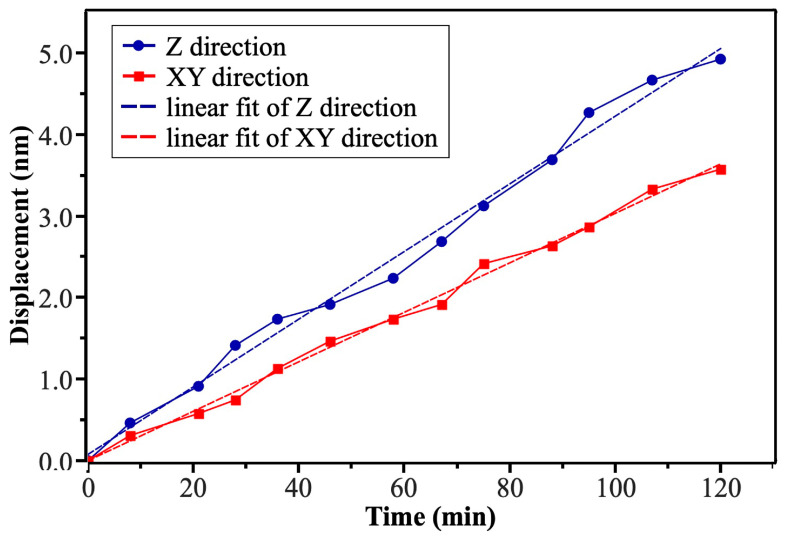
Measurements of drift rates as a function of time at 300 K in the cryogen-free magnet, showing 30.2 and 41.4 pm/min in the X–Y plane and Z direction by linear fitting, respectively.

To assess the long-term stability of the STM head, we measured the drift rates of the STM head in the X–Y plane and Z direction at 300K, as shown in Figure 4. By scanning a certain area of the HOPG sample continuously, we determined the lateral drift displacement over time. The drift displacement in the Z direction was measured by setting the tunneling current to 1 nA in the constant current mode and recording the voltage change in the scanning tube in the Z direction to convert it into a displacement amount. Through linear fitting of the measured two curves, we obtained the average drift rates of 30.2 pm/min and 41.4 pm/min in the X–Y plane and Z direction, respectively. The low drift rates illustrate the high stability of the instrument.

**Figure 5:**
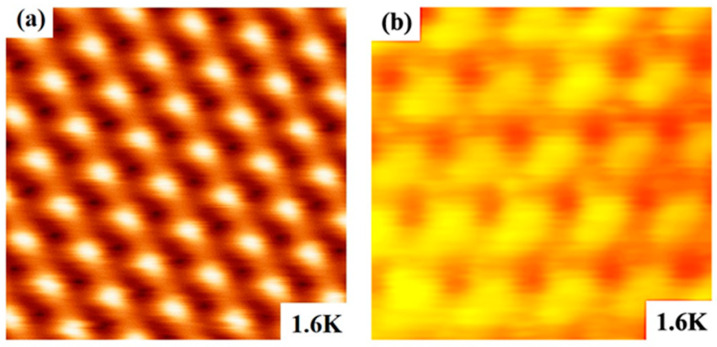
(**a**) Atomic-resolution STM image of HOPG obtained at 1.6 K. Scan area = 1.4 nm × 1.4 nm, bias voltage = + 200 mV, and average tunneling current = 10 nA. (**b**) Atomic-resolution STM image of NbSe_2_ obtained at 1.6 K. Scan area = 1.2 nm × 1.2 nm, bias voltage = + 100 mV, and average tunneling current = 5 nA.

To test the performance of the STM at low temperatures, we reduced the sample temperature to 1.6 K by controlling the flow through the VTI needle valve. Figure 5a shows an atomic-resolution image of the HOPG surface at 1.6 K. Figure 5b is an atomic-resolution image of a NbSe_2_ single crystal sample (from SixCarbon Technology); its surface is so sensitive to the atmosphere that it becomes unstable during imaging without in situ dissociation. The measured images show that the homemade STM also has excellent imaging performance at low temperatures.

**Figure 6:**
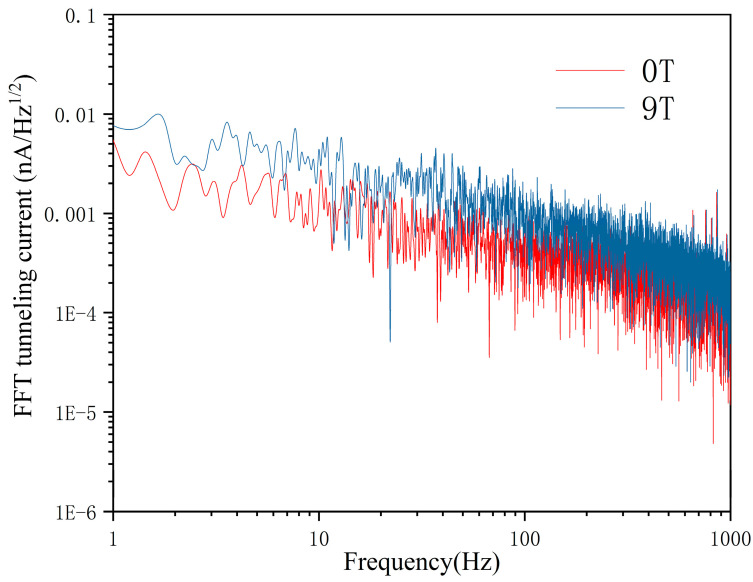
Spectral density plots of tunneling current measured in constant height mode at 1 nA. The plots in red and blue were obtained at 0 T and 9 T with a +200 mV bias voltage being applied on HOPG, respectively.

To characterize the noise level of a cryogen-free magnet STM system, we measured the tunneling current spectrums in constant height mode with and without magnetic fields, as shown in Figure 6. The noise from the pulse-tube cryocooler of the cryogen-free magnet is mainly in the low-frequency stage, which is consistent with the dominant peaks we observed, concentrated below 10 Hz. Under the field strength of 9 T, the increase in the current noise may be related to the electromagnetic interference generated by the magnet. However, owing to the attenuation of the low-frequency vibration noise by the two-stage damping of the STM rod, the overall noise level was still low enough to take the atomic-resolution imaging.

**Figure 7:**
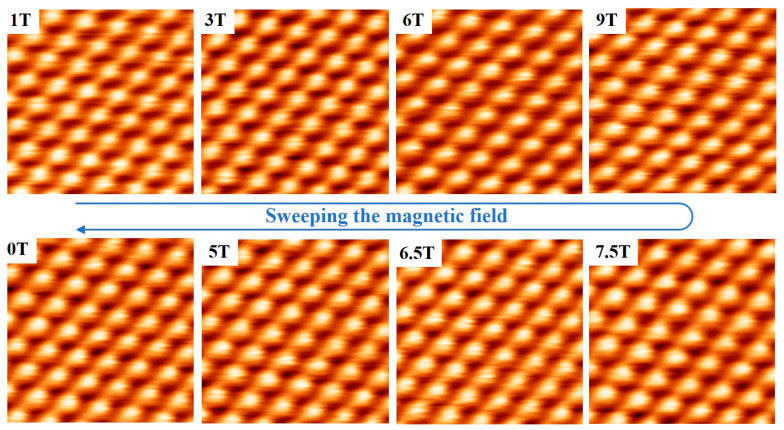
Continuous STM images of HOPG obtained in sweeping magnetic fields from 0 T to 9 T at 70 K with bias voltage +200 mV and average tunneling current 5 nA. The scan area is 1.5 nm × 1.5 nm.

Finally, the imaging ability of the STM in a magnetic field sweeping from 0 T to 9 T at 70 k was demonstrated, as shown in Figure 7. During the increased magnetic field, the atomic-resolution images of the HOPG at 1 T, 3 T, 6 T, and a high field of 9 T were obtained. The field reached 9 T and stayed at that strength for a while. Then, the field reduction stage was carried out, in which atomically resolved images were also obtained at 7.5 T, 6.5 T, 5 T, and 0 T. The imaging quality of the STM did not change significantly during the entire field variation process, indicating that our homemade improved STM is highly immune to high magnetic fields and external vibrations.

## 5. Conclusions

In summary, we reported on a new STM based on a cryogen-free superconducting magnet with a simple and compact STM head compatible with narrow sample spaces. The tip–sample loop was compact and not disturbed by the coarse approach motor. The atomic-resolution images obtained at 300 K and 1.6 K and low drift rate showed the STM’s excellent imaging performance and high stability. In the sweeping-field test from 0 T to 9 T, the high and stable atomic-resolution imaging quality of the pyrolytic graphite reflected our device’s high vibration resistance and immunity to high magnetic fields. Furthermore, the STM head’s short axial size gave it potential in a rotatable STM system to explore the modulation effect of in-plane magnetic fields on two-dimensional samples.

## Data Availability

The data presented in this study are available upon request from the corresponding author.
